# Chemosensory Gene Families in *Ectropis grisescens* and Candidates for Detection of Type-II Sex Pheromones

**DOI:** 10.3389/fphys.2017.00953

**Published:** 2017-11-21

**Authors:** Zhao-Qun Li, Zong-Xiu Luo, Xiao-Ming Cai, Lei Bian, Zhao-Jun Xin, Yan Liu, Bo Chu, Zong-Mao Chen

**Affiliations:** Key Laboratory of Tea Biology and Resource Utilization, Ministry of Agriculture, Tea Research Institute, Chinese Academy of Agricultural Science, Hangzhou, China

**Keywords:** transcriptomic analysis, chemoreception gene, sex pheromone perception, digital gene expression profiling, *Ectropis grisescens*, Type-II sex pheromone compounds, *Xenopus* oocytes

## Abstract

Tea grey geometrid (*Ectropis grisescens*), a devastating chewing pest in tea plantations throughout China, produces Type-II pheromone components. Little is known about the genes encoding proteins involved in the perception of Type-II sex pheromone components. To investigate the olfaction genes involved in *E*. *grisescens* sex pheromones and plant volatiles perception, we sequenced female and male antennae transcriptomes of *E*. *grisescens*. After assembly and annotation, we identified 153 candidate chemoreception genes in *E. grisescens*, including 40 odorant-binding proteins (OBPs), 30 chemosensory proteins (CSPs), 59 odorant receptors (ORs), and 24 ionotropic receptors (IRs). The results of phylogenetic, qPCR, and mRNA abundance analyses suggested that three candidate pheromone-binding proteins (EgriOBP2, 3, and 25), two candidate general odorant-binding proteins (EgriOBP1 and 29), six pheromone receptors (EgriOR24, 25, 28, 31, 37, and 44), and EgriCSP8 may be involved in the detection of Type-II sex pheromone components. Functional investigation by heterologous expression in *Xenopus* oocytes revealed that EgriOR31 was robustly tuned to the *E*. *grisescens* sex pheromone component (Z,Z,Z)-3,6,9-octadecatriene and weakly to the other sex pheromone component (Z,Z)-3,9-6,7-epoxyoctadecadiene. Our results represent a systematic functional analysis of the molecular mechanism of olfaction perception in *E*. *grisescens* with an emphasis on gene encoding proteins involved in perception of Type-II sex pheromones, and provide information that will be relevant to other Lepidoptera species.

## Background

In insects, chemical cues are regarded as language and play significant roles in regulating feeding, mating, and ovipositing (Zhou, 2010). Insect antennae, which are covered with several different types of chemosensory sensilla, are the principal chemosensory organs. Olfactory signal transduction starts with the recognition of odor molecules by olfactory receptors, such as odorant receptors (ORs) and ionotropic receptors (IRs) bound to olfactory receptor neuron (ORN) dendrites. However, the ORNs that are located within chemosensory sensilla are surrounded by aqueous lymphatic fluid. Thus, water-soluble carriers are required to transport lipophilic compounds through the sensilla lymph. Odorant-binding proteins (OBPs) and chemosensory proteins (CSPs) enhance the solubility of odors and deliver them to the olfactory receptors.

The OBPs of insects comprise ~150 amino acids and belong to the lipocalins superfamily (Flower, 1996), which comprises carrier proteins folded in the typical β-barrel structure (Tegoni et al., 2004). Their most striking feature is six highly conserved cysteines paired into three interlocked disulfide bridges (Pelosi et al., 2014). In the Lepidoptera (Gong et al., 2009), OBPs can be classified into pheromone-binding proteins/general odorant-binding proteins (PBPs/GOBPs), antennal binding protein I (ABPI), ABP II, chemical-sense-related lipophilic-ligand-binding protein (CRLBP), Minus-C, and Plus-C OBPs. The PBPs are thought to be involved in pheromone reception processes (Sun et al., 2013; Jin et al., 2014). The GOBPs are encoded by two paralogous genes (*GOBP1* and *GOBP2*) and are thought to be involved in the detection of plant volatiles and sex pheromones (Liu et al., 2015). The CSPs are soluble binding proteins that consist of 100–120 amino acid residues and have four conserved cysteines forming two independent loops (Angeli et al., 1999). Insects CSPs serve varied functions, including chemosensation (González et al., 2009) and development (Maleszka et al., 2007), as well as other processes (Kulmuni and Havukainen, 2013). For example, *Sesamia inferens* CSP19 and *Helicoverpa armigera* HarmCSP6 were reported to show high binding affinities for their respective sex pheromone components (Zhang et al., 2014a; Li et al., 2015).

Insect ORs play key roles in detecting odorants and triggering the transduction of chemical signals into electric signals (Spletter and Luo, 2009; Liu C. et al., 2013). Odorant receptor coreceptor (*ORco*) is one of the most highly conserved *OR* genes among various insect species (Nakagawa et al., 2012). It interacts with other ligand-specific ORs to form an OR–ORco complex, which functions as a ligand-gated cation channel (Leal, 2012). Pheromone receptors (PRs), a subfamily of ORs, are specifically activated by sex pheromone components and have been widely studied in Lepidopteran insects (Jiang et al., 2014; Zhang et al., 2014b; Chang et al., 2015). The IRs are an important and ancient repertoire of chemosensory receptors involved in olfaction (Benton et al., 2009) and gustation (Zhang et al., 2013). Previous studies have revealed that IRs also need a coreceptor (Benton et al., 2009). The proteins IR8a and IR25a are antennal IR coreceptors that are expressed at higher levels than other IRs in *Drosophila* and *Chilo suppressalis* (Rytz et al., 2013; Cao et al., 2014).

Tea grey geometrid, *Ectropis grisescens*, is a devastating chewing pest distributed in tea plantations throughout China. The sex pheromone components of *E. grisescens* have been characterized as (Z,Z,Z)-3,6,9-octadecatriene (Z3Z6Z9-18:Hy) and (Z,Z)-3,9-6,7-epoxyoctadecadiene (Z3Z9-6,7-epo-18:Hy) (Ma et al., 2016). Moth sex pheromone components can be divided into three types according to their structure: Type-I, Type-II, and miscellaneous type with proportions of 75, 15, and 10%, respectively (Ando et al., 2004). Type-I sex pheromone components comprise C_10_-C_18_ straight chain unsaturated alcohols, aldehydes, or acetate esters; and Type-II sex pheromone components consist of C_17_-C_23_ straight chains with 1–3 *cis* double bonds separated by methylene groups (Millar, 2000; Ando et al., 2004). Therefore, *E. grisescens* produces Type-II sex pheromone components. Most studies on the sex pheromone perception mechanism in Lepidopteran insects have focused on Type-I pheromone components (Jiang et al., 2014; Zhang et al., 2014b; Chang et al., 2015). Comparatively, little is known about Type-II pheromone components (Zhang et al., 2016). It is acknowledged and accepted that olfaction perception plays crucial roles in the chemical detection of *E. grisescens* (Sun et al., 2014; Ma et al., 2016). Thus, analysis of its olfactory molecular mechanism may identify targets for pest control. However, little is known about the molecular mechanisms regulating *E. grisescens* olfaction because of the paucity of sequence data for olfaction genes from *E. grisescens*. Therefore, to obtain such data as the primary step for exploring the olfaction mechanism, we constructed cDNA libraries of female and male antennae in *E. grisescens* and conducted several analyses to identify olfactory-related genes.

## Results

### Overview of antennae transcriptomes

**Figure 1:**
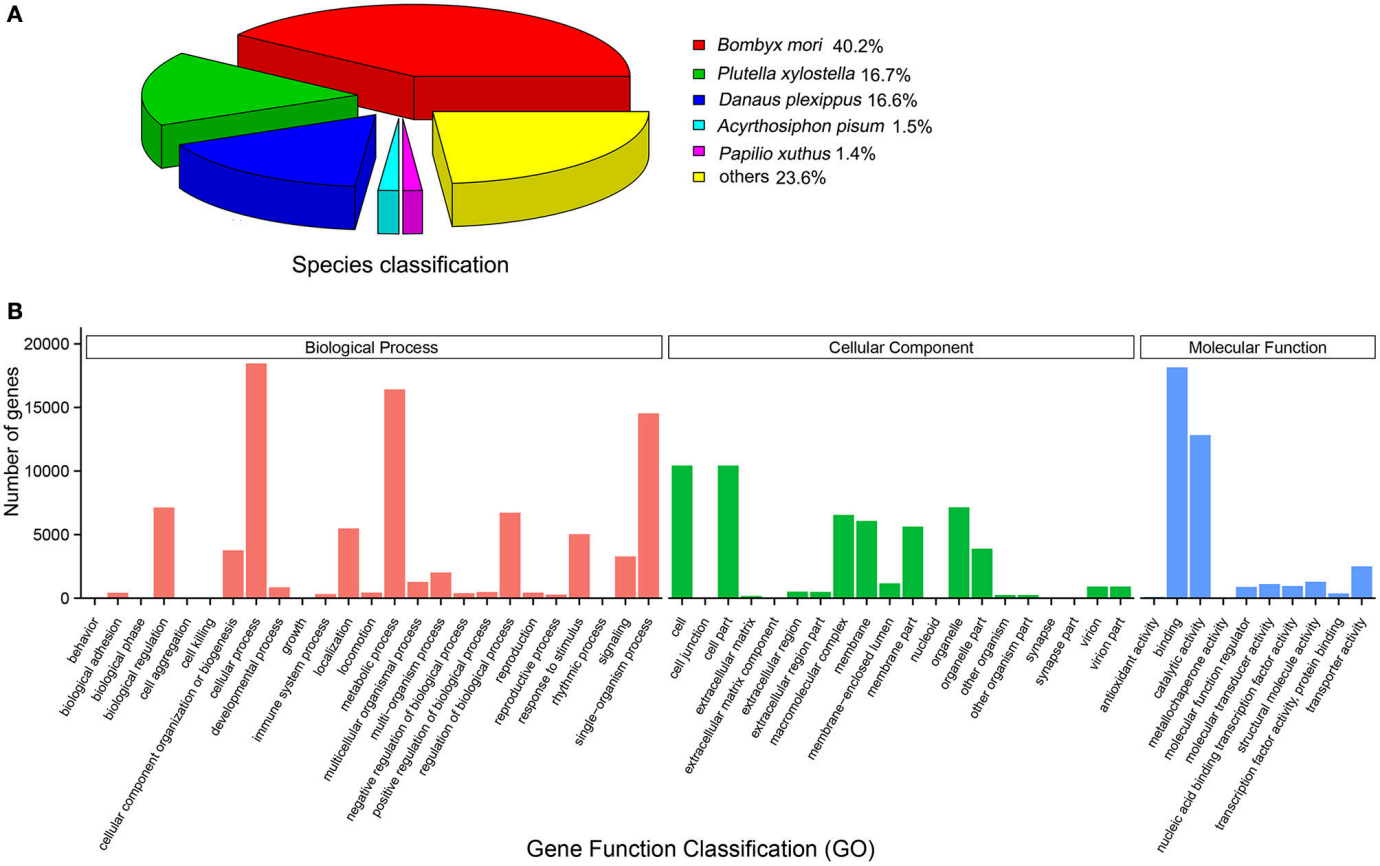
Annotation summaries for *E. grisescens* transcripts. **(A)** Species distribution of transcripts with best hit annotation terms in non-redundant (NR) database. **(B)** Gene ontology (GO) classifications of *E. grisescens* transcripts.

The transcriptomes of female antennae (FA) and male antennae (MA) of *E. grisescens* were sequenced with two independent biological replicates. About 45.72 (FA-1), 46.51 (FA-2), 42.15 (MA-1), and 49.97 (MA-2) million raw reads were obtained for each transcriptome. The datasets of transcriptomes during the current study are available in the NCBI SRA database (http://trace.ncbi.nlm.nih.gov/Traces/sra/, accession numbers: SRR6004297–SRR6004301). After filtering and assembling, 114,595 transcripts were generated with an N_50_ length of 1,715 nt (Table 1). For annotation, we combined the female- and male-antennal transcriptomes of *E. grisescens* and searched against the non-redundant (NR) database by BLASTX with a cut-off e-value of 10^−5^. The best match percentage (40.2%) was with *Bombyx mori* sequences, followed by sequences from *Plutella xylostella* (16.7%), *Danaus plexippus* (16.6%), *Acyrthosiphon pisum* (1.5%), and *Papilio xuthus* (1.4%) (Figure 1A). Gene ontology (GO) annotation was used to classify the transcripts into functional categories.

**Table 1:** *Ectropis grisescens* antennal transcriptome assembly and annotation.

	**FA-1**	**FA-2**	**MA-1**	**MA-2**
Total number of raw reads	45,722,672	46,510,102	42,151,920	49,965,856
Total number of clean reads	43,871,882	44,413,250	40,327,306	47,906,526
Clean bases	6.58 Gb	6.66 Gb	6.05 Gb	7.19 Gb
Total number Clean Nucleotides (nt)	6.58 Gb	6.66 Gb	6.05 Gb	7.19 Gb
Q_20_ percentage	97.51%	97.54%	97.26%	97.53%
GC percentage	40.19%	40.06%	39.68%	40.27%
Total number of transcripts	114,595			
N_50_ (nt)	1715			
Percentage of transcripts annotated by NCBI NR database	35.78%			
Percentage of transcripts annotated by Swiss-prot database	24.77%			
Percentage of transcripts annotated by PFAM database	28.34%			
Percentage of transcripts annotated by KOG database	17.8%			
Percentage of transcripts annotated by GO database	28.54%			

Among the distinct transcripts, 32,710 (28.54%) corresponded to at least one GO term.

Within the molecular function category, most transcripts were involved in binding (e.g., nucleotide-, ion-, and odorant-binding) and catalytic activity (e.g., hydrolase and oxidoreductase) (Figure 1B).

### Identification of *E. grisescens* OBP/CSP/OR/IR genes and sequence analyses

**Figure 2:**
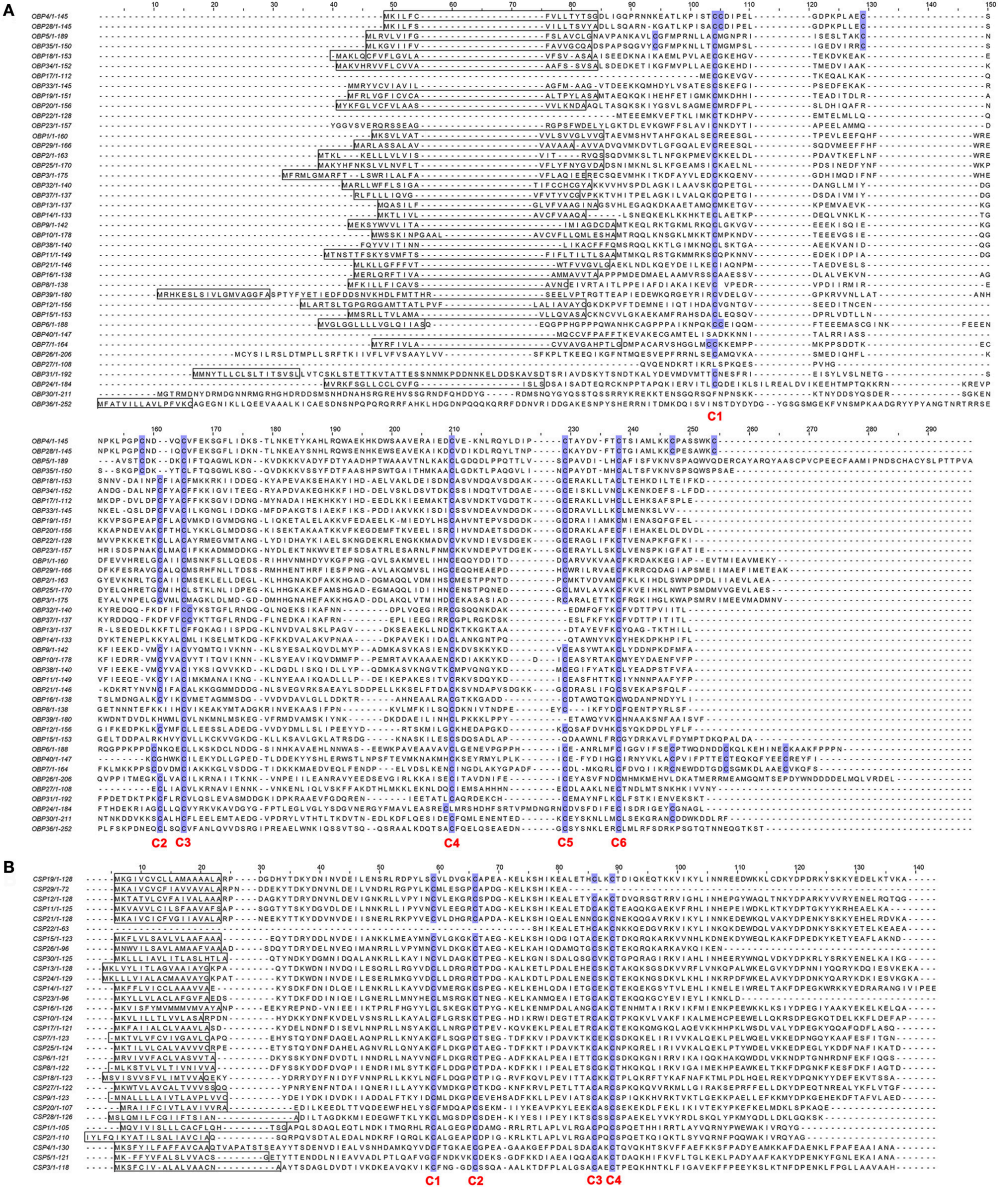
Alignment of amino acid sequences of EgriOBPs and EgriCSPs. **(A)** Alignment of amino acid sequences of the EgriOBPs. **(B)** Alignment of amino acid sequences of EgriCSPs. Boxes indicate predicted signal peptides, blue highlight indicates conserved cysteines.

Sequence annotation led to the identification of 40 candidate *EgriOBPs* in the *E. grisescens* antennae transcriptome (File S1). Sequence analyses showed that 34 of them were full-length *EgriOBP* genes, and 31 had a predicted signal peptide (Figure 2A). EgriOBP13, 14, 15, 32, 37, and 39 contained four conserved cysteines but lacked the conserved cysteines C2 and C5. EgriOBP4, 5, 6, 7, 28, 35, and 40 contained more than six conserved cysteines. EgriOBP8 contained five conserved cysteines but lacked the conserved cysteine C2. All of the *E. grisescens* classic OBPs present the C-pattern common to Lepidoptera, C1-X_25−30_-C2-X3-C3-X_36−42_-C4-X_8−14_-C5-X_8_-C6. A total of 30 *CSP* genes were identified in *E. grisescens* antennae, 26 of which contained a full-length open reading frame (ORF), a signal peptide, and four conserved cysteine residues (Figure 2B). All of the *E. grisescens* CSPs have the C-pattern of Lepidoptera, C1-X_6−8_-C2-X_18_-C3-X_2_-C4. By homology analysis, we identified 59 candidate *EgriORs* in *E. grisescens* antennae. Sequence analyses revealed that 45 (*EgriOR1–44* and *EgriORco*) of the 59 sequences had an intact ORF with characteristics typical of insect *OR* genes (full-length ORFs of about 1,200 bp). In total, we identified 24 *EgriIRs* from *E. grisescens* antennal transcriptomes, and 16 of them contained intact ORFs.

### Phylogenetic analyses

**Figure 3:**
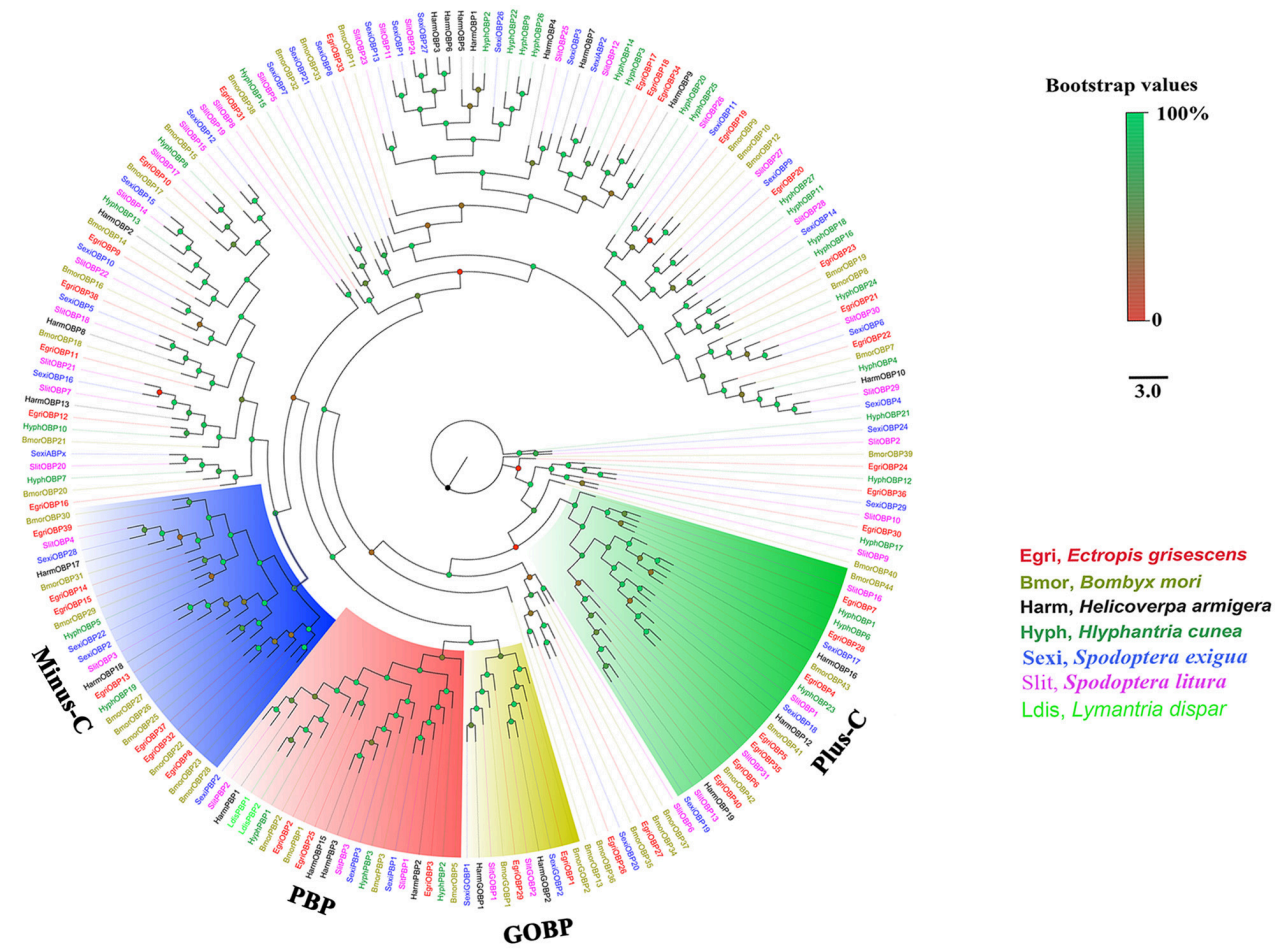
Phylogenetic analysis of EgriOBPs with other typical insect OBPs. Phylogenetic tree was constructed in PhyML3.0 using maximum likelihood method.

**Figure 4:**
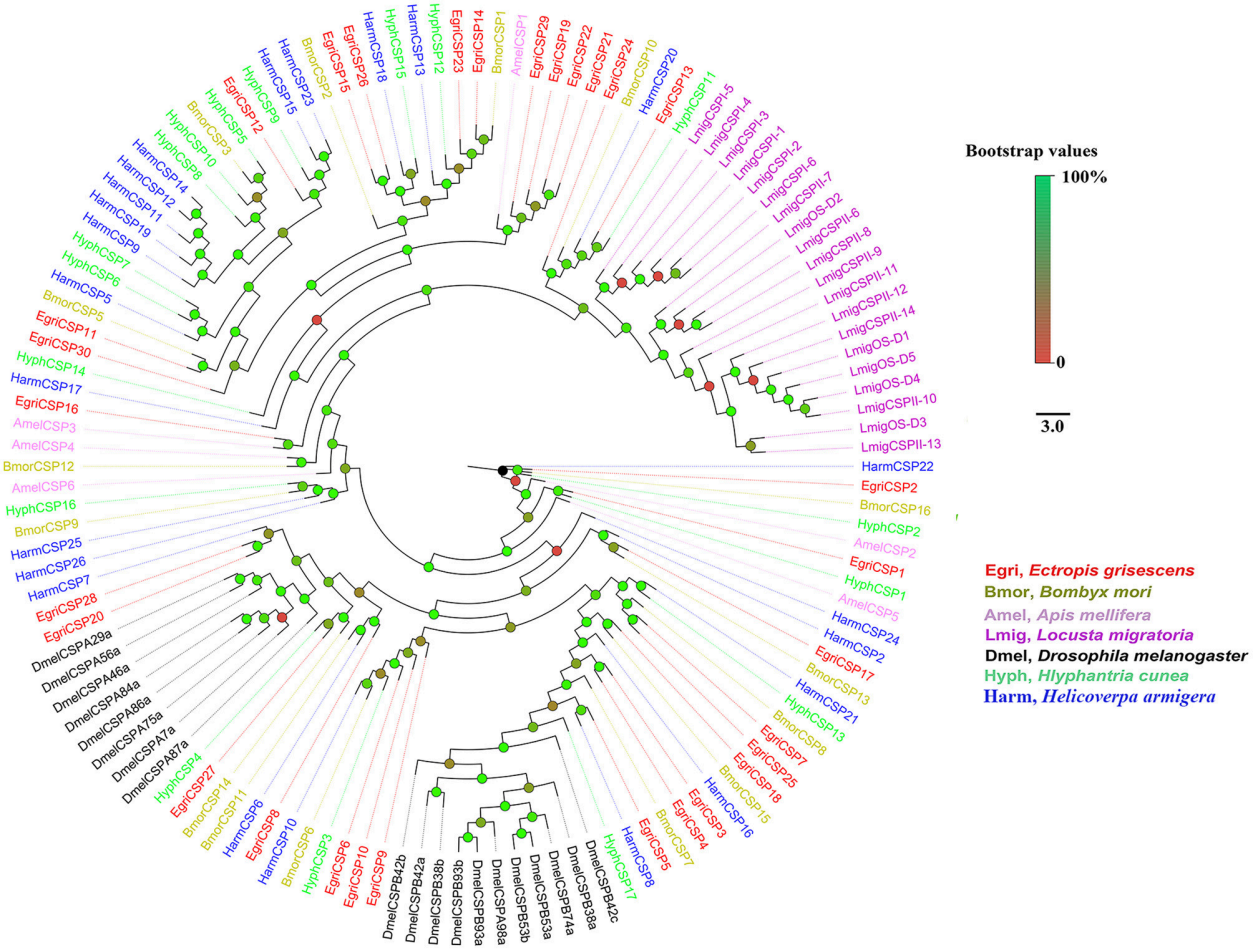
Phylogenetic analysis of EgriCSPs with other typical insect CSPs. Phylogenetic tree was constructed in PhyML3.0 using maximum likelihood method.

To further investigate the function of *E. grisescens OBP/CSP/OR/ IR* genes, phylogenetic trees were constructed using sequences of typical OBP/CSP/OR/IRs from other insect species for which the whole genome or transcriptome data were available. In the resulting phylogenetic tree, we observed four well-supported groups; PBP, GOBP, Plus-C OBP, and Minus-C OBP (Figure 3). Three EgriOBPs (EgriOBP2, 3, and 25) were grouped in the PBP clade with another Lepidoptera PBP. The orthologs EgriOBP1 and 29 were in the GOBP group. EgriOBP4, 5, 6, 7, 28, 35, and 40 were distributed in the Plus-C OBP group, and EgriOBP8, 13, 14, 15, 32, 37, and 39 were distributed in the Minus-C OBP group. In the CSP phylogenetic tree, EgriCSP8 was grouped into the same clade as HarmCSP6 (Figure 4).

**Figure 5:**
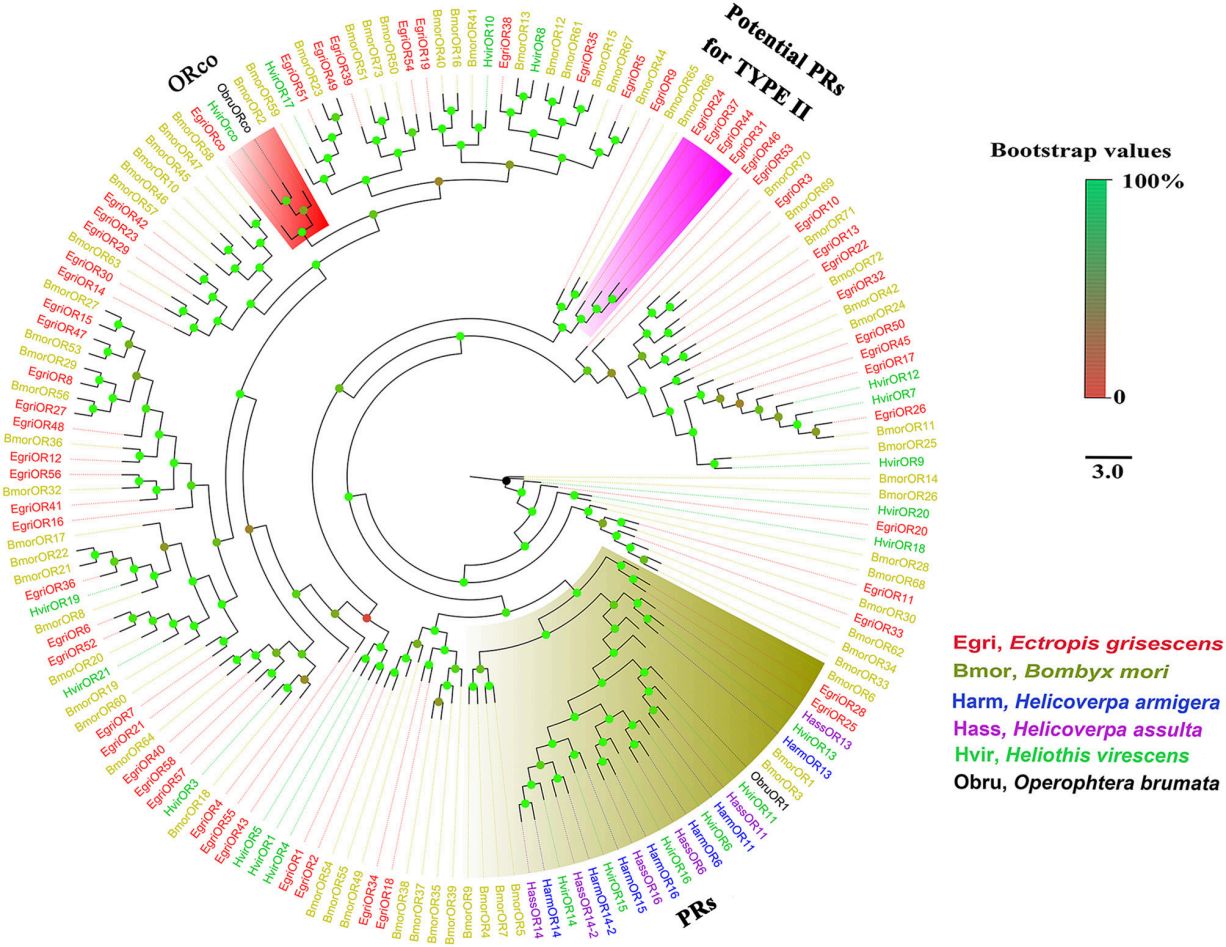
Phylogenetic analysis of EgriORs with other typical insect ORs. Phylogenetic tree was constructed in PhyML3.0 using maximum likelihood method.

**Figure 6:**
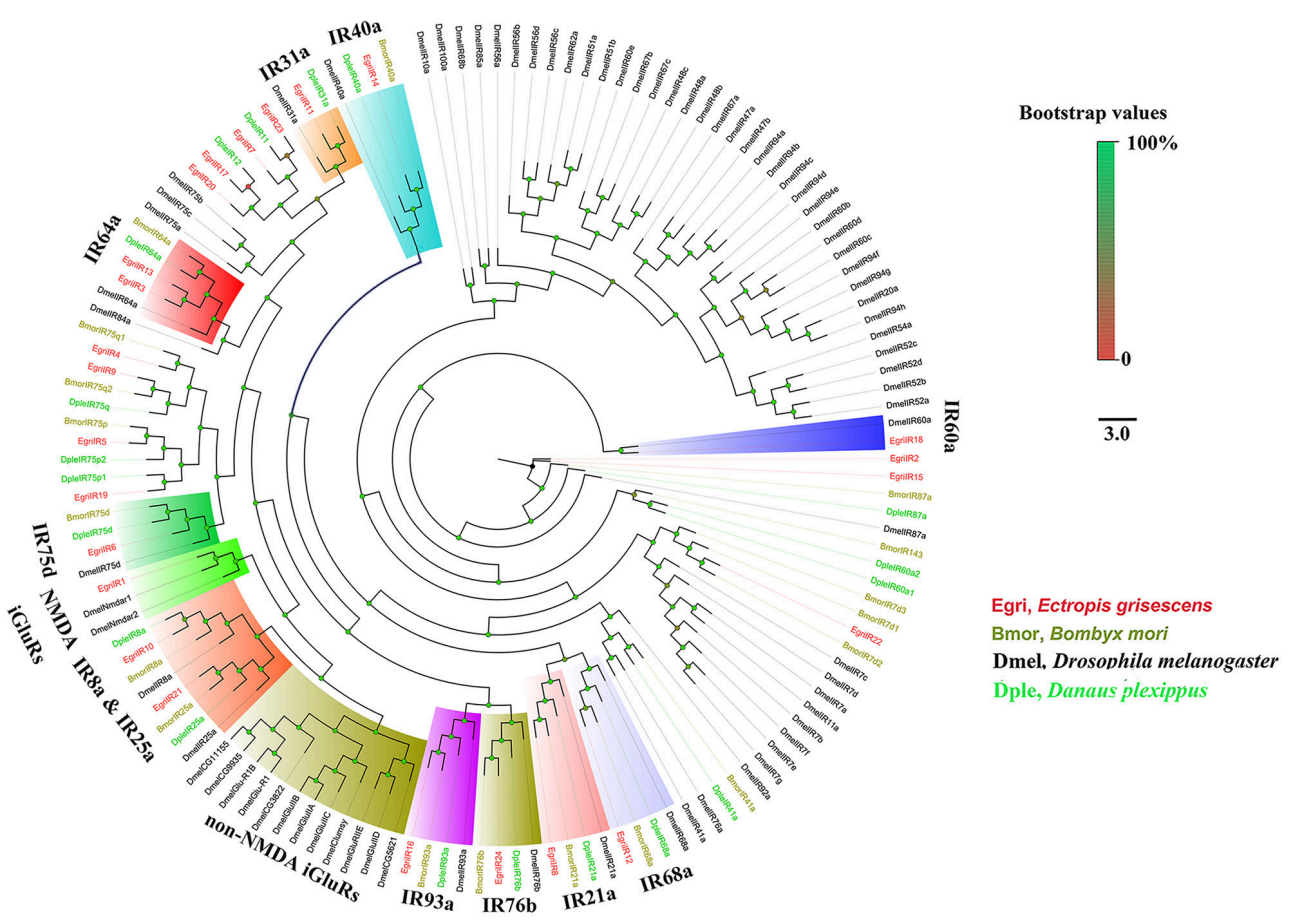
Phylogenetic analysis of EgriIRs with other typical insect IRs. Phylogenetic tree was constructed in PhyML3.0 using maximum likelihood method.

The EgriORs were distributed among the orthologous groups in the OR phylogenetic tree (Figure 5). EgriORco was strongly associated with ObruORco, HvirORco, and BmorOR2 with high bootstrap support. EgriOR25, 28, and ObruOR1 were grouped with *B. mori, H. armigera, Helicoverpa assulta*, and *Heliothis virescens* PRs, which are known to be receptors for Type-I pheromones. EgriOR24, 31, 37, and 44 were independently grouped without any orthologs of other Lepidoptera insects. In the IR phylogenetic tree (Figure 6), 11 EgriIRs (Egri3, 6, 8, 11, 12, 13, 14, 16, 18, and 24) were clustered with the presumed “antennal” orthologs IR64a, IR21a, IR31a, IR68a, IR75d, IR76b, IR93a, IR60a, and IR40a. Two EgriIRs, EgriIR10, and EgriIR21, were respectively distributed in the IR8a and IR25a groups, which are co-receptors. EgriIR1 was grouped with NMDA iGluRs (N-methyl-d-aspartate ionotropic receptors).

### Tissue expression profile and mRNA abundance of *E. grisescens* OBP/CSP/OR/IR genes

**Figure 7:**
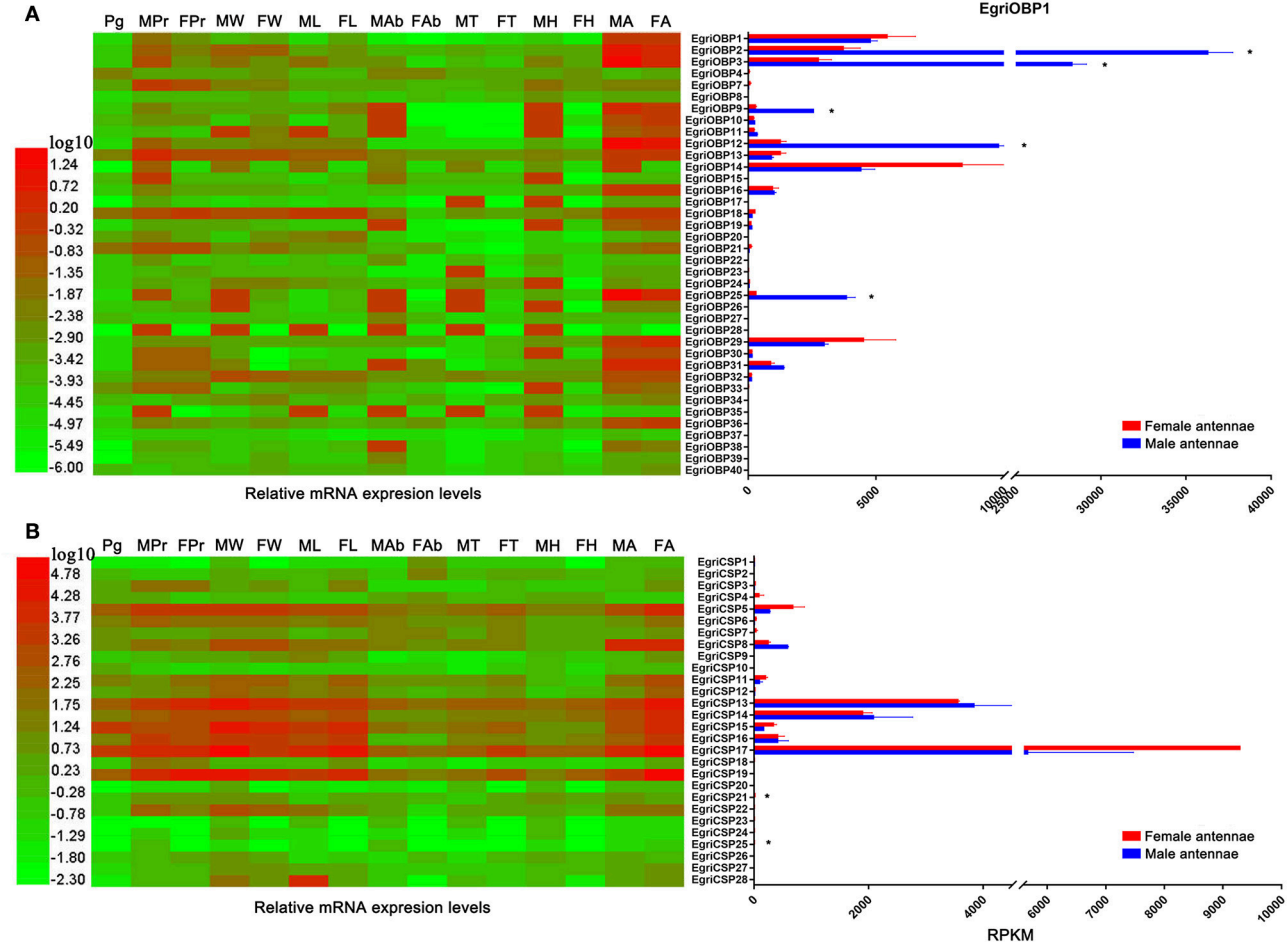
Tissue expression profiles and abundance of selected *EgriOBP* and *EgriCSP* genes in antennae based on relative mRNA quantity and RPKM values. Heat map illustrates Log_10_-transformated mRNA expression levels of *EgriOBP* and *EgriCSP* in different tissues. Histogram shows RPKM values of *EgriOBP* and *EgriCSP*. **(A)** Tissue expression profile and RPKM values of selected *EgriOBP* genes. **(B)** Tissue expression profile and RPKM values of selected *EgriCSP* genes. FA, female antennae; MA, male antennae; FH, female head without antennae; MH, male head without antennae; FT, female thorax; MT, male thorax; FAb, female abdomen without pheromone glad; MAb, male abdomen; FL, female legs; ML, male legs; FW, female wings; MW, male wings; FPr, female proboscis; MPr, male proboscis; Pg, pheromone gland. *P-value < 0.05.

We further characterized the tissue expression pattern and abundance of *E. grisescens OBP/CSP/OR/IR* genes in the antennae by qPCR and by evaluating the RPKM (reads per kilobase per million mapped reads) values. The qPCR results indicated that 24 *EgriOBP*s were uniquely or more strongly expressed in female and male antennae, except for *EgriOBP4, 7, 8, 15, 21, 22, 23, 24, 27, 28, 35, 37, 39*, and *40* (Figure 7A). Of these 24 *EgriOBPs* showing antenna-biased expression, *EgriOBP2, 3, 9, 12*, and *25* showed significantly higher transcript levels in male antennae than in female antennae (9.8-, 10.3-, 9.0-, 7.8-, and 12.8-fold higher RPKM values, respectively, in male antennae than in female ones). Of the five male antenna-biased *EgriOBPs, EgriOBP2* and *3* were much more abundant in the antennae transcriptome. *EgriOBP7, 13, 21*, and *33* transcripts were abundant mainly in female and male proboscises. Compared with *EgriOBPs, EoblCSPs* showed wider and more diverse expression patterns (Figure 7B). The transcript levels of *EgriCSP5, 8, 13, 14, 15, 16*, and *17* were markedly higher in the *E. grisescens* antennae transcriptomes than in the transcriptomes of other tissues. Of these seven *EgriCSPs* that were abundant in the antennae, *EgriCSP8* was expressed at higher levels in male antennae than in female ones. The abundance of *EgriCSP21* and *25* transcripts was markedly higher in female antennae than in male ones. The abundance levels of EgriCSP5, 15, and 17 were also higher in female antennae than in male ones, but the differences were not significant.

**Figure 8:**
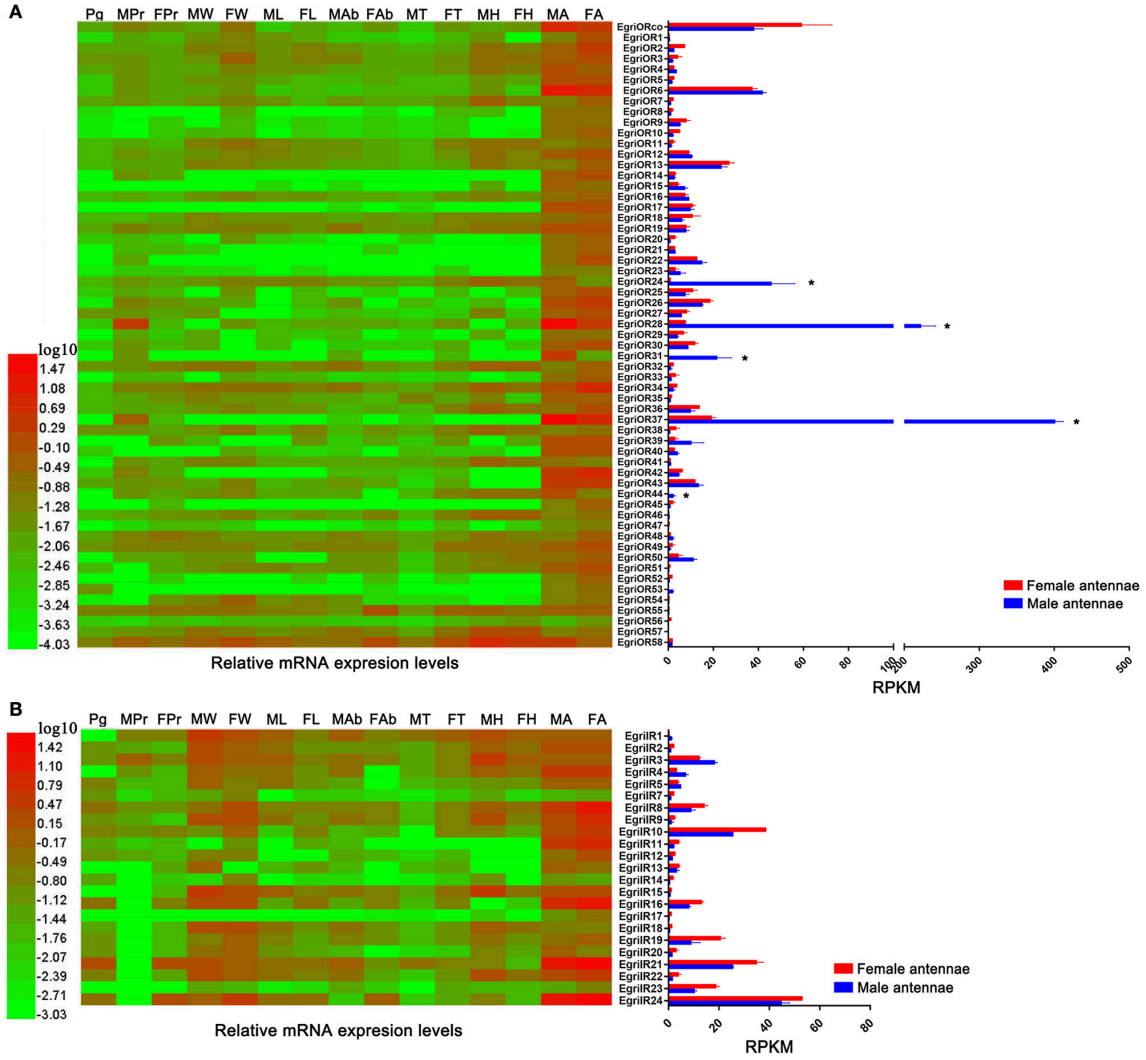
Tissue expression profiles and abundance of selected *EgriOR* and *EgriIR* genes in antennae based on relative mRNA quantity and RPKM values. Heat map illustrates Log_10_-transformed mRNA expression levels of *EgriOR* and *EgriIR* in different tissues. Histogram shows RPKM values of *EgriOR* and *EgriIR*. **(A)** Tissue expression profile and RPKM values of selected *EgriOR* genes. **(B)** Tissue expression profile and RPKM values of selected *EgriIR* genes. FA, female antennae; MA, male antennae; FH, female head without antennae; MH, male head without antennae; FT, female thorax; MT, male thorax; FAb, female abdomen without pheromone gland; MAb, male abdomen; FL, female legs; ML, male legs; FW, female wings; MW, male wings; FPr, female proboscis; MPr, male proboscis; Pg, pheromone gland. *P < 0.05.

Analyses of the expression profile of *EgriORs* showed that these *EgriOR* genes were uniquely or more strongly expressed in antennae than in other tissues (Figure 8A). Among the *ORs*, including *EgriORco, EgriOR28* and *37* showed the highest expression levels in antennae. Five *EgriORs* (*EgriOR24, 28, 37, 44*) were predominantly expressed in male antennae, with RPKM values in male antennae being 51.6-, 29.6-, 20.9-, and 72.7-fold that of their respective counterparts in female antennae. *EgriOR31* was uniquely expressed in male antennae. Analyses of the expression patterns of *EgriIR* genes indicated that *EgriIR8, 10, 11, 12, 16, 21*, and *24* were highly expressed in the antennae (Figure 8B). Of these eight *EgriIRs, EgriIR10, 21*, and *24* showed relatively high RPKM values in female and male transcriptomes.

### Functional study of EgriOR31

**Figure 9:**
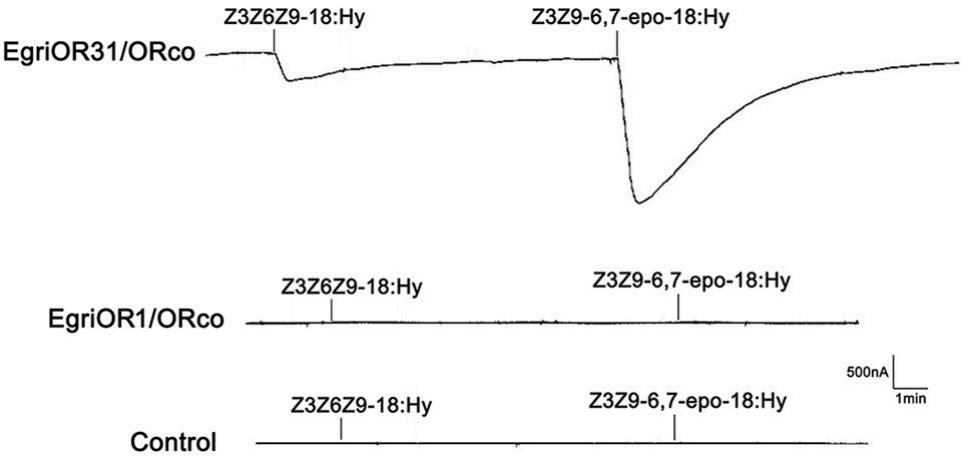
Responses of *Xenopus* oocytes co-expressing EgriOR31/EgriORco and EgriOR1/EgriORco to stimulations with pheromone compounds. Inward current responses of EgriOR31/EgriORco and EgriOR1/EgriORco *Xenopus* oocytes in response to 10^−5^ M solutions of sex pheromone compounds.

The *Xenopus* oocytes and two-electrode voltage clamping recording system were used to characterize the function of the EgriOR1 and 31, by co-expressing with the corresponding receptor EgriORco. The results showed that oocytes co-expressing EgriOR31 and EgriORco robustly responded to the triene Z3Z9-6,7-epo-18:Hy, but less so to Z3Z6Z9-18:Hy. The oocytes co-expressing EgriOR1 and EgriORco showed no responses (Figure 9).

## Discussion

Chemical cues, including sex pheromones and host plant volatiles, drive several aspects of insect behavior, such as mating, feeding, and selection of oviposition sites. Sex pheromone-induced behaviors play crucial roles in insect reproduction. The sex pheromone components of *E. grisescens* are Z3Z6Z9-18:Hy and Z3Z9-6,7-epo-18:Hy (Ma et al., 2016), both of which are Type-II compounds. However, little is known about the molecular mechanisms regulating *E. grisescens* olfaction. Therefore, we analyzed the antennal transcriptomes of female and male *E. grisescens* to identify genes involved in the perception of sex pheromones and host plant volatiles. In our study, we sequenced *E. grisescens* female and male antennal transcriptomes, with two independent biological replicates. A total of 26.48 Gb of antennae transcriptome data was obtained. Sequence assembly yielded 114,595 transcripts from *E. grisescens* antennal transcriptomes. After annotation, we identified 153 candidate chemosensory genes (40 EgriOBPs, 30 EgriCSPs, 59 EgriORs, and 24 EgriIRs) in *E. grisescens* (File S1).

Insect PBPs represent a sub-class of OBPs that are specifically dedicated to the detection of sex pheromones (Zhou, 2010). *EgriOBP2* and *3* were the two most abundant *EgriOBPs* in the antennal transcriptome with ~10-fold higher RPKM values in male antennae than in female ones. *EgriOBP25* showed a relatively high RPKM value in male antennae. The phylogenetic tree showed that EgriOBP2, 3, and 25 were distributed in the PBP group with PBPs from *S*. *inferens* (Jin et al., 2014), *Spodoptera exigua* (Liu et al., 2014), *Spodoptera litura* (Liu N. Y. et al., 2013), *H*. *armigera* (Dong et al., 2017), and *Hlyphantria cunea* (Sanes and Plettner, 2016). That is, *EgriOBP2, 3*, and *25* were expressed at higher levels in male antennae than in female ones, they were more abundant than other OBPs in male antennae, and they showed homology to other insect PBPs that are known to function in sex pheromone binding. Therefore, *EgriOBP2, 3* and *25* may encode PBPs for Type-II pheromone components. Aside from *EgriOBP2, 3*, and *25, EgriOBP9* and *12* also showed significantly higher expression in male antennae than in female ones, with relatively high RPKM values in male antennae. However, they were not homologous to PBPs of other Lepidoptera insects that produce Type-I pheromone components. Further research is required to clarify the roles of EgriOBP9 and 12 in sex pheromone perception in *E. grisescens*.

Another key sub-class of OBPs, the GOBPs, are known to be sensitive to both sex pheromones and plant volatiles (Liu et al., 2015). EgriOBP1 and 29 were distributed in GOBP clade. Of them, EgriOBP1 was grouped in the GOBP2 sub-classed with *S. litura* (Liu et al., 2015), *B. mori* (Zhou et al., 2009), and *S. exigua* (Liu et al., 2014) GOBP2 which could strongly bind sex pheromones. Consequently, we speculate that EgriOBP1 may be involved in the binding of sex pheromone components in *E. grisescens*.

Based on their cysteine motifs, inset OBPs can be classified into several groups: classical OBPs (with six conserved cysteines), Minus-C (with four conserved cysteines) (Forêt and Maleszka, 2006), Plus-C OBPs (with more than six conserved cysteines) (Hekmat-Scafe et al., 2002; Zhou et al., 2004), and dimer OBPs (which contain two amino acid sequence domains) (Hekmat-Scafe et al., 2002). Several Minus-C OBPs (*H*. *armigera* HarmOBP17 and 18 and *Apis mellifera* OBP 14) show high binding affinities to plant volatiles (Spinelli et al., 2012; Li et al., 2013). In our study, EgriOBP14 and EgriOBP13 were distributed in the same cluster with HarmOBP17 and HarmOBP18, respectively. Thus, these two EgriOBPs might have similar functions to HarmOBP17 and 18, and play roles in plant volatiles perception. EgriOBP22 was associated with *H*. *armigera* HarmOBP10, which is expressed at high levels in both antennae and seminal fluid and may function as a carrier of oviposition deterrents (Sun et al., 2012).

Insect PRs, a key sub-class of ORs, are responsible for detecting sex pheromone components in the Lepidoptera (Jiang et al., 2014; Zhang et al., 2014b; Chang et al., 2015). Due to the lack of data for PRs for Type-II pheromone components, we constructed the phylogenetic tree using PRs for Type-I pheromone components. Out of 59 EgriORs, two (EgriOR25 and 28) were grouped in the PR clade with PRs for Type-I sex pheromone components. Among them, *EgriOR28* was predominantly expressed in male antennae, with an RPKM value 29.6-fold higher in male antennae than in female antennae. *EgriOR28* was the second most abundant OR in antennae. Therefore, these two *EgriORs*, particularly *EgriOR28*, may encode PRs for Type-II sex pheromone components. Generally, Lepidoptera insects have about 5~6 PRs; for example, six PRs were identified in both *H*. *armigera* (Liu et al., 2012) and *C*. *suppressalis* (Cao et al., 2014). On the other hand, Geometroidea species that produce Type-II sex pheromones, including *E. grisescens*, are more highly evolved than species that produce Type-I sex pheromones. Therefore, we speculate that another EgriORs involved in the detection of sex pheromones in *E. grisescens* might be distributed in a group separate from that containing PRs for Type-I sex pheromone components.

In fact, four EgriORs (EgriOR24, 31, 37, and 44) formed an independent group in the phylogenetic analysis. In addition, all four of these *EgriORs* showed higher abundance in male antennae than in female ones (RPKM values in the male antennae being 51.6-, 29.6-, 20.9-, and 72.7-fold that of their respective counterparts in female antennae). Among these four male antenna-biased *EgriORs, EgriOR37* was the most abundant *EgriOR* in antennal transcriptomes, and *EgriOR24* and *31* showed relatively high RPKM values in male antennae. Consequently, it is conceivable that EgriOR24, 31, 37, and 44 might be potential PRs for Type-II sex pheromone components. To test this above hypothesis, we respectively co-expressed EgriOR31 and EgriOR1 (an EgriOR that sorted to a different phylogenetic clade with EgriOR24, 31, 37, and 44) with the corresponding co-receptor EgriORco, and tested it against the sex pheromone of *E. grisescens*. The results showed that EgriOR31 and EgriORco robustly responded to Z3Z9-6,7-epo-18:Hy and weakly to Z3Z6Z9-18:Hy. However, EgriOR1 and EgriORco showed no responses. This result indicated that these four male antennae abundant EgriORs (EgriOR24, 31, 37, and 44) which formed an independent group in the phylogenetic analysis might also be potential PRs for Type-II sex pheromone components.

The CSPs and IRs are known to be involved in insect odorant reception. In the CSP phylogenetic tree, EgriCSP8 grouped in the same clade as HarmCSP6, which is responsible for the perception of sex pheromones (Li et al., 2015). In addition, *EgriCSP8* showed a male antenna-biased expression pattern with a relatively high RPKM value in male antennae, suggesting that EgriCSP8 plays a role in *E. grisescens* sex pheromone detection. Consistent with the roles of IRs in olfaction, most of the EgriIRs were clustered with “antennal” orthologs and displayed high expression levels in olfactory tissues. The IRs are known to detect acids, amines, and other odorants that are not sensed by ORs (Benton et al., 2009; Ai et al., 2010, 2013). Of the 24 EgriIRs, EgriIR10, 21, and 24, which was grouped in the antennal IR group, showed relatively high RPKM values in female and male transcriptomes. These results indicated that it might play key roles in olfaction, especially in male *E. grisescens*.

In conclusion, we sequenced the female and male antennae transcriptomes of *E. grisescens* to identify the genes involved in chemoreception, with an emphasis on genes encoding proteins involved in the perception of Type-II sex pheromone components. The results of phylogenetic, gene expression, and transcript abundance analyses indicate that a number of EgriOBPs, EgriORs, and EgriCSPs with male antenna-biased expression could be involved in sex pheromone detection. In particular, EgriOR24, 31, 37, and 44 might be potential PRs for Type-II sex pheromone components. Functional investigation revealed that EgriOR31 was tuned to the *E*. *grisescens* sex pheromone components.

## Materials and methods

### Insect rearing and tissue collection

Individuals of *E. grisescens* were originally collected from the Experimental Tea Plantation of the Tea Research Institute, Chinese Academy of Agricultural Sciences (Hangzhou, Zhejiang, China). Experimental insects were reared on fresh tea shoots in enclosed nylon mesh cages (70 × 70 × 70 cm) and kept in a climate-controlled room at 25 ± 1°C and 70 ± 5% relative humidity under a 14-h light:10-h dark photoperiod. After pupation, male and female pupae were kept separately in cages for eclosion. After emergence, adult moths were supplied with 10% honey solution. For transcriptome sequencing, 100 female and 100 male antennae were collected from 2-day-old virgin insects, with two replicates. Female and male antennae, heads, thoraxes, thoraxes, legs, wings, proboscises, and pheromone glands were collected from 3-day-old virgin insects for qRT-PCR analyses. These tissues were immediately frozen and stored at −80°C until RNA isolation.

### cDNA library preparation and illumina sequencing of transcriptomes

Total RNA was extracted from female and male antennae using TRIzol reagent (Invitrogen, Carlsbad, CA, USA). Quality of the RNA were assessed by agarose gel electrophoresis, Nanodrop (Thermo), and Agilent 2100. The sampling quality satisfy the requirements of cDNA libraries construction. The cDNA library construction and Illumina sequencing were performed at Novogene Bioinformatics Technology Co., Ltd. (Beijing, China). Briefly, mRNAs were isolated from 5 μg pooled total RNA using oligo (dT) magnetic beads and fragmented into short fragments in the presence of divalent cations in fragmentation buffer at 94°C for 5 min. Using these short fragments as templates, random hexamer primers were used to synthesize first-strand cDNA. Second-strand cDNA was generated using RNase H, and DNA polymerase I. After end repair and adaptor ligation, short sequences were amplified by PCR and purified with a QIAquick® PCR purification kit (Qiagen, Venlo, The Netherlands), and sequenced on the HiSeq™ 2500 platform (San Diego, CA, USA).

### *De novo* assembly of short reads and functional annotation

Transcriptome *de novo* assembly was carried out with the short-read assembly program Trinity (r20140413p1) (Grabherr et al., 2011) based on the paired-end reads with default settings. Transcripts longer than 150 bp were first aligned by BLASTX to protein databases (NR, Swiss-Prot, KEGG, and COG; *e*-value < 10^−5^) to retrieve proteins with the highest sequence similarity to the unigenes along with their protein functional annotations. We then used the Blast2GO (Conesa et al., 2005) for GO annotation of the transcripts and WEGO software (Ye et al., 2006) to plot the GO annotation results.

### Analysis of transcript expression in the antennal transcriptomes

Transcript abundance was calculated by the RPKM (reads per kilobase per million mapped reads) method (Mortazavi et al., 2008), which can eliminate the influence of different transcript lengths and sequencing discrepancies when calculating abundance (Mortazavi et al., 2008). We used the following equation:

$$RPKM(A)={\frac{C\times 10}{\frac{N\times L}{10^{3}}}}^{6}$$

where *RPKM (A)* is the RPKM value of transcript *A*; *C* is the number of reads uniquely aligned to transcript *A*; *N* is the total number of fragments uniquely aligned to all transcripts; and *L* is the number of bases in transcript *A*.

### Differential expression analysis

Genes showing differential expression between two conditions/groups were detected using the DESeq R package (1.10.1), which provides statistical routines to determine differential expression from digital gene expression data using a model based on negative binomial distribution. The resulting *P* values were adjusted using Benjamini and Hochberg's approach to control the false discovery rate. Genes with an adjusted *P* < 0.05 found by DESeq were designated as being differentially expressed.

### Sequence alignment and phylogenetic analysis

Amino acid sequence alignments of the candidate 40 EgriOBPs and 30 EgriCSPs were performed using ClustalX 2.0 (Larkin et al., 2007), and visualized using Jalview 2.4.0 b2 (Waterhouse et al., 2009). The signal peptides of EgriOBPs and EgriCSps were predicted by SignalP 4.1 (http://www.cbs.dtu.dk/services/SignalP/). To investigate the phylogenetic relationships of OBPs, CSPs, ORs, and IRs between *E. grisescens* and other typical insect species, we compared them using MAFFT (E-INS-I parameter) (Katoh and Standley, 2013). Phylogenetic trees were constructed using PhyML 3.1 with LG substitution model was used to construct a maximum likelihood phylogenetic tree using Bayesian analysis (Guindon et al., 2010).

### Quantitative real-time PCR validation

The tissue expression patterns of 40 *OBP*s, 30 *CSP*s, 59 *OR*s, and 24 *IR*s in different tissues were measured by a qPCR method conducted according to the minimum information for publication of quantitative Real-Time PCR Experiments (Bustin et al., 2009). Total RNA was isolated using the SV Total Isolation System (Promega, Madison, WI, USA) according to the manufacturer's instructions. Quality of the RNA were assessed by agarose gel electrophoresis, Nanodrop (Thermo). Single-stranded cDNA templates were synthesized using the Reverse Transcription System (Promega) as per the manufacturer's instructions. The qRT-PCRs were performed on a Bio-Rad CFX96 touch real-time PCR detection system (Bio-Rad, Hercules, CA, USA). The primers were designed using Beacon Designer 7.7 based on the *E. grisescens* nucleotide sequences obtained from the transcriptome data (File S2). Templates diluted into five-fold series were used to construct a relative standard curve to determine the PCR efficiencies and for quantification analysis. Each reaction was run in triplicate (technical repeat). Two reference genes, guanine nucleotide-binding protein G(q) subunit alpha and glyceraldehyde-3-phosphate dehydrogenase (sequences were provided in File S3), were selected in qPCR analysis. A blank control without template cDNA (replacing cDNA with H_2_O) served as the negative control. Each reaction included three independent biological replicates and was repeated three times (technical replicates). Relative transcript levels were calculated using the comparative 2^−ΔΔ^Cq method. The level of each tested mRNA was determined using SYBR® Premix Ex Taq™ II (TaKaRa, Dalian, China) according to the manufacturer's instructions.

### Functional study of EgriOR31

A *Xenopus* oocytes expression system was used to express the EgriOR1 and 31. EgriOR1 and 31 were amplified using specific primers (File S2). The purified PCR products were ligated into pT7Ts vector using an In-Fusion® HD Cloning Kit (Clontech, USA) following manufacturer's instructions. The cRNAs of EgriORs were synthesized using mMESSAGE Mmachine T7 kit (Ambion, Austin, TX). Electrophysiological recording was performed according to previously reported protocols (Wang et al., 2010). Mature healthy oocytes (stage V–VII) were treated with 2 mg/ml collagenase I(GIBCO, Carlsbad, CA) in washing buffer [96 mM NaCl, 2 mM KCl, 5 mM MgCl_2_, and 5 mM HEPES (pH = 7.6)] for about 1.5 h at room temperature. And then, oocytes were microinjected with 27.6 ng EgriOR cRNAs and 27.6 ng EgriORco. After injection, oocytes were incubated for 4–7 days at 18°C in 1 × Ringer's solution [96 mM NaCl, 2 mM KCl, 5 mM MgCl_2_, 0.8 mM CaCl_2_, and 5 mM HEPES (pH = 7.6)] supplemented with 5% dialyzed horse serum, 50 mg/ml tetracycline, 100 mg/ml streptomycin, and 550 mg/ml sodium pyruvate. Whole-cell currents were recorded from the injected *Xenopus* oocytes with a two-electrode voltage clamp and recorded with an OC-725C oocyte clamp (Warner Instruments, Hamden, CT, USA). Stock solutions of tested compounds were prepared using ethyl alcohol, which were diluted to the indicated concentrations by 1 × Ringer's buffer before use. Oocytes were exposed to1 × 10^−5^ M of sex pheromone compounds and ethyl alcohol. Oocytes without cRNA injection were set as negative control. Data acquisition and analyses were carried out with Digidata 1440A and pCLAMP 10.2 software (Axon Instruments Inc., Union City, CA).

## Author contributions

Z-QL and Z-MC conceived and designed the experiments; Z-QL performed the experiments; Z-QL, Z-XL, X-MC, LB, Z-JX, YL, and BC analyzed the data; and Z-QL wrote the manuscript. All authors reviewed the final manuscript.

### Conflict of interest statement

The authors declare that the research was conducted in the absence of any commercial or financial relationships that could be construed as a potential conflict of interest.

## Supplementary material

File S2 **Primers used in qPCR and Vector construction**.

File S3 **Sequences of guanine nucleotide-binding protein G(q) subunit alpha and glyceraldehyde-3-phosphate dehydrogenase**.
