# Supplementary material for: Chemosensory Gene Families in Ectropis grisescens and Candidates for Detection of Type-II Sex Pheromones
Source: Front Physiol. 2017 Nov 21;8:953. doi: 10.3389/fphys.2017.00953 (PMC5702326; doi:10.3389/fphys.2017.00953)
Supplement: File S3 — Sequences of guanine nucleotide-binding protein G(q) subunit alpha and glyceraldehyde-3-phosphate dehydrogenase. [file DataSheet3.DOCX]

>glyceraldehyde-3-phosphate dehydrogenase

ATGTCGAAGATCGGAATTAACGGATTTGGCCGTATCGGCCGTCTCGTCCTCCGCGCCTCCATCGAGAAGGGCGCCCAGGTGGTCGCCATCAACGACCCCTTCATCGGACTCGACTACATGGTCTACCTCTTCCAGTACGACTCCACCCACGGCCGCTTCAAGGGCACCGTCTCCGTCGTCGATGGCCACCTCGTCGTAAACGGCAACAAAATCGCCGTCTTCTCCGAACGCGACCCCAAAGCCATCCCGTGGGGCAAGGCTGGCGCCGAGTACGTCGTCGAATCCACCGGCGTGTTCACCACCATCGACAAAGCTTCCGCTCACTTGGAAGGTGGAGCCAAGAAGGTCATCATCTCCGCTCCCAGCGCTGACGCCCCCATGTTCGTCGTTGGCGTCAACCTCGAAGCTTACGATCCCTCTCACAAGGTCATCTCCAACGCTTCGTGCACCACCAACTGCCTGGCGCCCCTCGCCAAGGTCATCCATGACAACTTTGAGATTGTTGAAGGTCTGATGACCACTGTGCACGCCACCACTGCCACCCAGAAGACCGTAGATGGACCCTCCGGAAAGCTGTGGCGTGATGGCCGTGGCGCTCAGCAGAACATCATTCCAGCGTCCACTGGCGCGGCTAAGGCCGTAGGCAAAGTCATTCCCGCTCTTAACGGAAAGCTGACTGGTATGGCTTTCCGTGTCCCCGTGGCCAACGTATCCGTCGTCGACCTCACTGTCCGCCTTGGCAAGCCTGCCTCCTACGATGCCATCAAACAGAAGGTCAAGGAAGCCGCTGAGGGACCCCTTAAGGGTATCCTCGGATACACTGAAGACCAGGTCGTATCTTCCGACTTCATTGGTGACTCGCACTCGTCCATCTTCGATGCTGCTGCTGGTATCTCTCTGAACGACAACTTCGTCAAACTCATCAGCTGGTATGATAACGAATATGGCTACTCTAGCCGTGTCATCGATCTCATCAAGTACATCCAGACCAAGGATTAA

>guanine nucleotide-binding protein G(q) subunit alpha

ATGGAGTGCTGCATGTCAGAAGAGGCCAAAGAACAGAAAAGAATCAATCAGGAAATAGAGAGACAACTCCGGAAGGACAAACGAGATGCCAGGAGAGAACTCAAGTTGCTGCTACTCGGCACTGGCGAGTCAGGCAAGTCGACATTCATCAAGCAGATGAGAATCATCCACGGCTCCGGCTACAGCGACGATGACAAGCGCGGCTTCATCAAGCTCGTCTACCAAAACATCTTCATGGCCATGCAAAGTATGATACGGGCCATGGAACTACTCAGTATACAATACGGGATACCGTCCAATGCAGAAAAGGCGGAGTTAATATCAGCTATCGACTTCGAGAGTGTTACGACGTTCGACAGTCCGTACGTAGAAGCTATAAAGGCGCTGTGGTCCGACGCTGGTATCCAGGAGTGCTACGATCGCAGACGAGAGTACCAACTCACTGACTCAGCCAAATACTACTTGCAAGAGATTGACCGCGTGGCGGCACCAAATTACTTACCGACCGAACAAGACATCCTCCGTGTGAGAGTGCCCACAACGGGCATCATCGAATACCCCTTCGATTTGGAGGAGATTCGATTTCGAATGGTGGATGTCGGGGGCCAAAGATCCGAGCGAAGGAAGTGGATTCACTGTTTCGAAAACGTCACCTCTATCATATTCTTAGTAGCTCTTAGTGAATATGATCAAATTTTATTCGAATCAGAAAATGAGAACCGAATGGAAGAGTCGAAGGCGCTATTCAAGACGATCATCACGTACCCCTGGTTCCAGCATTCGTCTGTAATCCTCTTCCTCAACAAGAAGGATTTGCTTGAAGAGAAGATCATGTATTCGCATCTCGTTGATTACTTCCCTGAATATGACGGTCCCCAGCGCGACGCGAATACGGCGCGCGAGTTCATCCTCCGAATGTTTGTGGACCTCAATCCGGACGCTGAGAAGATTATCTACTCGCATTTCACCTGCGCGACGGACACCGAGAACATCCGGTTCGTGTTCGCCGCAGTTAAGGACACCATCCTACAGTCCAACCTGAAGGAGTACAACCTGGTCTAA
